# Research trends and hotspots of infertility and phthalate esters: a bibliometric and visualization analysis from 2001 to 2024

**DOI:** 10.3389/fmed.2025.1563179

**Published:** 2025-08-29

**Authors:** Xiaoyue Jiang, Xinyuan Liu, Fei Luo, Yinyin Ding, Lu Bai, Shangyuan Liu, Huijin Zhao, Bei Liu, Huifang Zhou

**Affiliations:** ^1^Nanjing University of Chinese Medicine, Nanjing, China; ^2^Department of Gynecology, Affiliated Hospital of Nanjing University of Chinese Medicine, Nanjing, China; ^3^Department of Pediatrics, Affiliated Hospital of Nanjing University of Chinese Medicine, Nanjing, China; ^4^Department of Otorhinolaryngology, Affiliated Hospital of Nanjing University of Chinese Medicine, Nanjing, China

**Keywords:** PAEs, infertility, bibliometric analysis, visualization analysis, hotspots, frontiers

## Abstract

**Background:**

Phthalate esters (PAEs) are a class of synthetic compounds that are extensively utilized in a range of consumer products, and they significantly affect both environmental conditions and human reproductive health. Especially, many studies have been conducted to investigate the association between PAEs and infertility. However, a bibliometric analysis on this topic has yet to be published.

**Objective:**

To conduct a bibliometric and visualization analysis of published articles concerning the association between PAEs and infertility, in order to identify and assess the research trends and hotspots in the field of infertility and PAEs, with a particular focus on by analyzing the temporal shift in exposure pathways and their impact on key unresolved aspects: mixed exposures, dose–response relationships, and toxicological mechanisms.

**Methods:**

The Web of Science Core Collection (WoSCC) database was searched for publications on infertility and PAEs between 2001 and 2024, to collect the authors, institutions, countries, references, keywords and impact factor (IF). CiteSpace 6.3.1, VOSviewer 1.6.20 and Bibliometrix Biblioshiny 4.1 were utilized to perform this bibliometric and visualization analysis.

**Results:**

This study analyzed a total of 406 documents, including 322 articles and 84 reviews. The authors and institutions with the largest number of publications belong to China and the USA. Reproductive Toxicology and Environment International have published the highest number of articles, and three most frequently cited journals mainly involve the fields of environmental health perspectives, human reproduction, and toxicological sciences. The analysis of keyword co-occurrence and explosive words shows that the keywords with the highest frequency are “bisphenol a,” “infertility” and “testicular dysgenesis syndrome,” “endocrine disrupting chemicals,” “oxidative stress” and “bisphenol a,” “infertility” are the research focuses and hotspots in recent years, which provides valuable guidance for PAEs and infertility from the aspects of etiology, pathogenesis, diagnosis and treatment.

**Conclusion:**

These results serve to elucidate the research hotspots associated with PAEs and infertility. Furthermore, it assists researchers in concentrating on contemporary research trends and offering guidance for future research, particularly in the areas of etiology, pathological mechanisms, diagnosis, and treatment.

## 1 Introduction

Phthalate esters (PAEs), recognized as prevalent plasticizers, represent a category of synthetic organic compounds characterized by significant production volumes and extensive applications globally (1). These compounds are extensively utilized across various sectors, including medical devices, cosmetics, construction materials, toys, and food packaging, to enhance the flexibility and ductility of plastics (2). Worldwide production of PAEs is approximately 8 million tons annually, and it is anticipated that PAEs account for 80–85% of the overall types of plasticizers produced (3, 4).

PAEs possess endocrine-disrupting properties and are classified as typical environmental endocrine disruptors. While they exhibit a degree of stability, these substances can easily be released and subsequently migrate into the atmosphere, aquatic systems, and soil over time. This situation not only facilitates human activities and production processes but also poses a significant threat to the ecological environment and human reproductive health (5–7). These compounds can interfere with the endocrine system, adversely affecting reproductive development and neuroimmune systems of organisms, thereby contributing to an elevated risk of obesity (8, 9), hypertension (10), diabetes (11), pregnancy and postpartum anxiety and depression symptoms (12). Furthermore, exposure to PAEs can induce dysfunction in the hypothalamus-pituitary-gonadal (HPG) axis, resulting in abnormal secretion of gonadal hormones and alterations in the synthesis of sex hormone receptors (13–15). This dysregulation may lead to detrimental effects, including oxidative stress and apoptosis, which may ultimately undermine reproductive health, exemplified by testicular damage (16) and reduced fertility capacity (17).

Moreover, approximately 15% of the global adult population experiences infertility, which constitutes a significant public health concern (18). Recent research indicates a decline in human reproductive health and the increase in infertility in industrialized regions, the phenomenon that has been associated with the presence of PAEs and chemicals derived from fossil fuels (19). However, there exists a limited number of bibliometric analysis concerning PAEs and infertility. A bibliometric analysis of published articles will aid in summarizing and assessing current research findings, offering more useful insights for understanding and preventing infertility caused by PAEs.

The bibliometric analysis involves the application of quantitative methods to systematically analyze and evaluate the existing literature within particular domains, specifically focusing on performance analysis and visual analysis (20). Utilizing visualization analysis software enables an intuitive examination of the significance and interconnections of publications within a relational network (21–23). This approach facilitates the analysis of advancements in specific academic disciplines and can forecast their potential developmental trajectories and evolutionary pathways (24).

Therefore, to address the existing gaps in the current research, of PAEs and infertility, we employed comprehensive bibliometric analysis to evaluate the publications published in the Web of Science (WOS) database by utilizing software like CiteSpace 6.3.1, VOSviewer 1.6.20 and Bibliometrix Biblioshiny 4.1. We hope this approach systematically delineates the evolutionary trajectory, research trends and hotspots within this field, specifically focuses on the temporal changes in exposure pathways and their impact on key unresolved questions, particularly those related to mixed exposures, dose-response relationships, and toxicological mechanisms.

## 2 Materials and methods

### 2.1 Data source and search strategy

The Web of Science Core Collection^1^ encompasses a diverse array of publications from various disciplines and features a robust citation index capability that can track the citation relationship between documents (25). So it can provide comprehensive and high-quality academic resources for bibliometrics research.

In order to clarify publications of the relationship between Infertility and PAEs, the search strategy selected was TS = (infertility OR sterility OR dysgenesis OR acyesis OR dysgenesia OR aciesis OR barreness OR barren OR barrenness OR infecundity OR infertile OR “infertile disease” OR “non-pregnancy”) AND TS = (PAEs OR “phthalic acid ester” OR “phthalic ester” OR phthalate OR PAE OR “phthalate ester” OR “dimethyl phthalate esters” OR “diethyl phthalate” OR “dioctyl phthalate” OR “dibutyl phthalate” OR “dimethyl phthalate”), with a time span starting from January 2001 to October 2024 by screening the Web of Science Core Collection (WoSCC). The database was set to SSCI and SCI-Expanded. Publications categorized as Articles and Review Articles in English-language was considered, resulting in 526 records. Information regarding the search terms can be found in Table 1.

**Table 1:** The information of data source and search strategy.

Category	Specific standard requirements
Research database	Web of Science core collection
Citation indexes	Science Citation Index Expanded (SCI-EXPANDED), Social Sciences Citation Index (SSCI)
Searching period	January 1, 2001 to October 31, 2024
Language	English
Searching keywords	TS = (infertility OR sterility OR dysgenesis OR acyesis OR dysgenesia OR aciesis OR barreness OR barren OR barrenness OR infecundity OR infertile OR “infertile disease” OR “non-pregnancy”) AND TS = (PAEs OR “phthalic acid ester” OR “phthalic ester” OR phthalate OR PAE OR “phthalate ester” OR “dimethyl phthalate esters” OR “diethyl phthalate” OR “dioctyl phthalate” OR “dibutyl phthalate” OR “dimethyl phthalate”)
Subject categories	All
Document types	“Articles” OR “Review Articles”
Data extraction	Export with full records and cited references in plain text format
Sample size	526

Further inclusion and exclusion criteria were established for the selection of relevant literature. The inclusion criteria encompassed original research articles (Articles), which include human observational studies and experiments conducted on mammalian models, as well as review articles (Review Articles). Conversely, the exclusion criteria eliminated non-English publications, studies that did not measure exposure to PAEs, and publications that did not concentrate on reproductive health outcomes, such as those that solely analyzed environmental concentrations.

**Figure 1:**
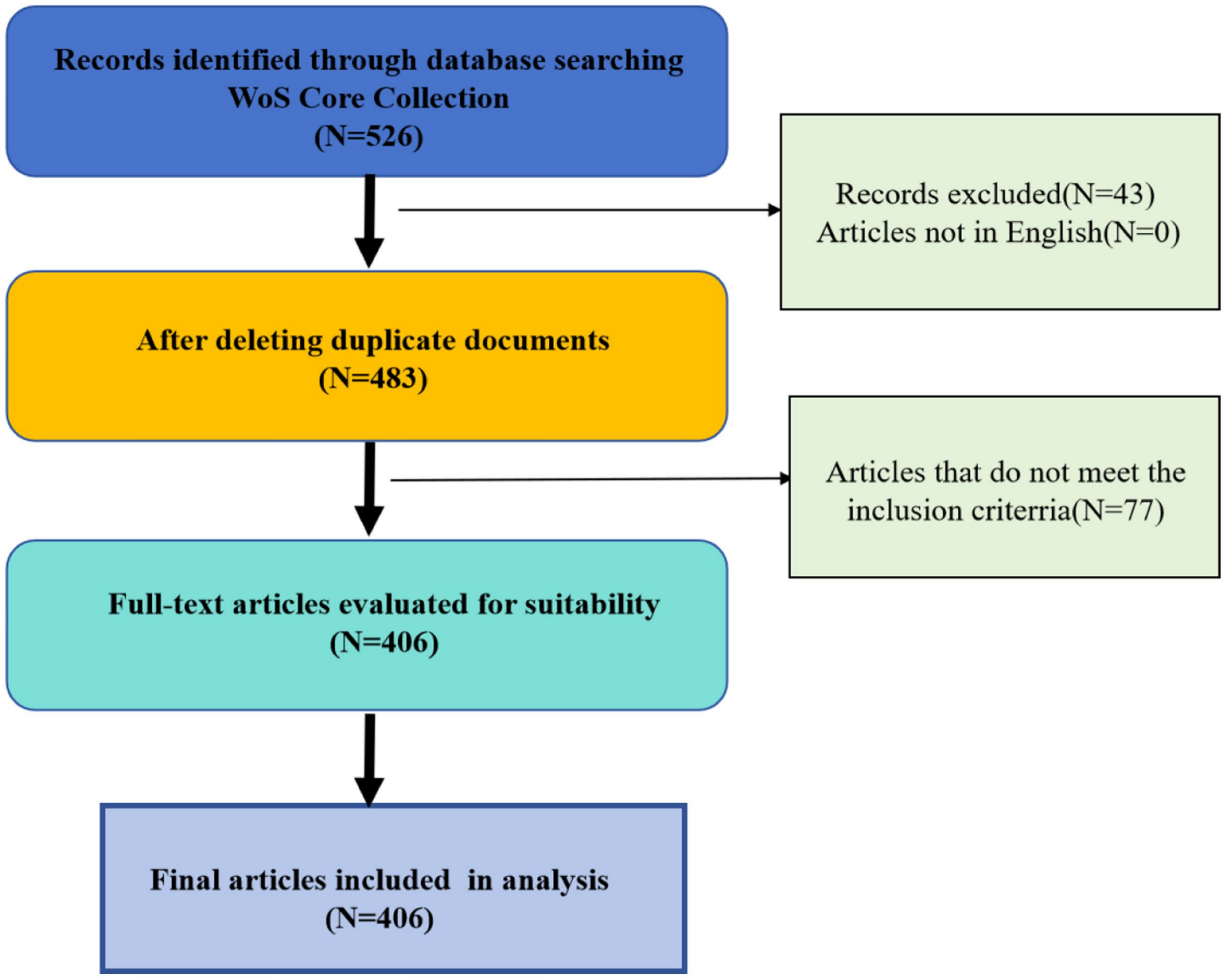
Flowchart of documents selection.

After two researchers (Jiang XY and Liu XY) addressed any disputes and relevance inconsistencies, evaluated and screened each article, 406 valid publications were ultimately selected. The flowchart illustrating the process of publications selection is shown in Figure 1.

### 2.2 Data analysis and visualization

According to the standard, the key information of WOS documents is filtered and exported to Microsoft Excel 2021, including authors, journals, countries, institutions, references, keywords, impact factor (IF) and so on. VOSviewer (version 1.6.20) was used to visualize authors, journals, countries, institutions, references and keywords, including Network Visualization, Overlay Visualization and Density Visualization. Keywords that are either duplicated or contain relationships are merged. For example, the keywords “di ethylhexyl-phthalate” or “di-(2-ethylhexyl) phtalate (DEHP),” “n butyl phthalate” or “dibutyl phthalate” or “di-n-butyl Phthalate (DBP),” “di-n-hexyl phthalate (DNHP),” “diethyl phthalate (DEP),” “di isobutyl phthalate (DIBP)” and “phthalate” are unified into “phthalate esters”; “bisphenol,” “bisphenol-a,” “BPA” are unified into “bisphenol a,” “endocrine disrupters,” “endocrine disruption” and “environmental chemicals” are unified into “endocrine disrupting chemicals.” By utilizing online platform Bibliometrix Biblioshiny 4.1^2^ to analyze the publications and compute the H index, we can gain a deeper insight into and evaluate the academic contributions and impact of researchers. Then, we analyze timeline view, keywords clusters by Citespace (version 6.3.1), the development and historical timeline of research on “PAEs” and “infertility” are mapped out, and new research trends in this field are highlighted.

## 3 Results

### 3.1 Analysis of the annual trends in publications

**Figure 2:**
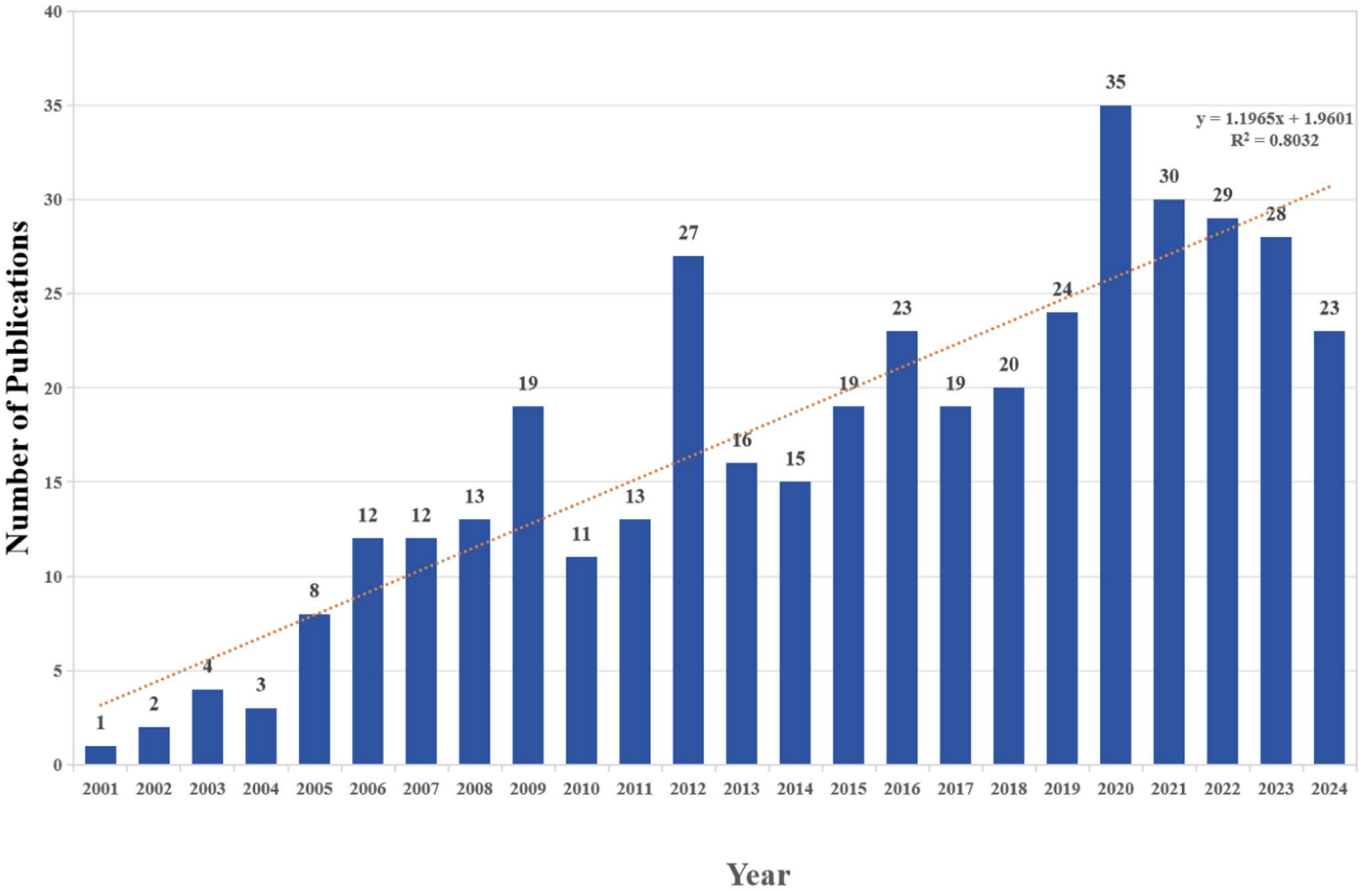
The number of annual publications relating to research on PAEs and infertility from 2001 to 2024.

This study analyzed a total of 406 publications, comprising 322 articles and 84 reviews, authored by 1990 individuals from 596 organizations across 48 countries. These papers were published in 165 journals and cited 16,780 references. Among the non-review publications (Articles), epidemiological studies accounted for 53%, while experimental studies accounted for 47%. We created a histogram (Figure 2) to illustrate the annual trends in publications related to research on PAEs and infertility from 2001 to 2024, highlighting the growth in this area of study. The polynomial function is expressed as y = 1.1965x + 1.9601, (*R*^2^ = 0.8032). Overall, the number of publications in this field has been increasing, particularly after 2015, indicating a growing interest among researches in recent years. The year 2020 represents the highest number of publications, with a total of 35 articles.

### 3.2 Contributions of the authors

**Figure 3:**
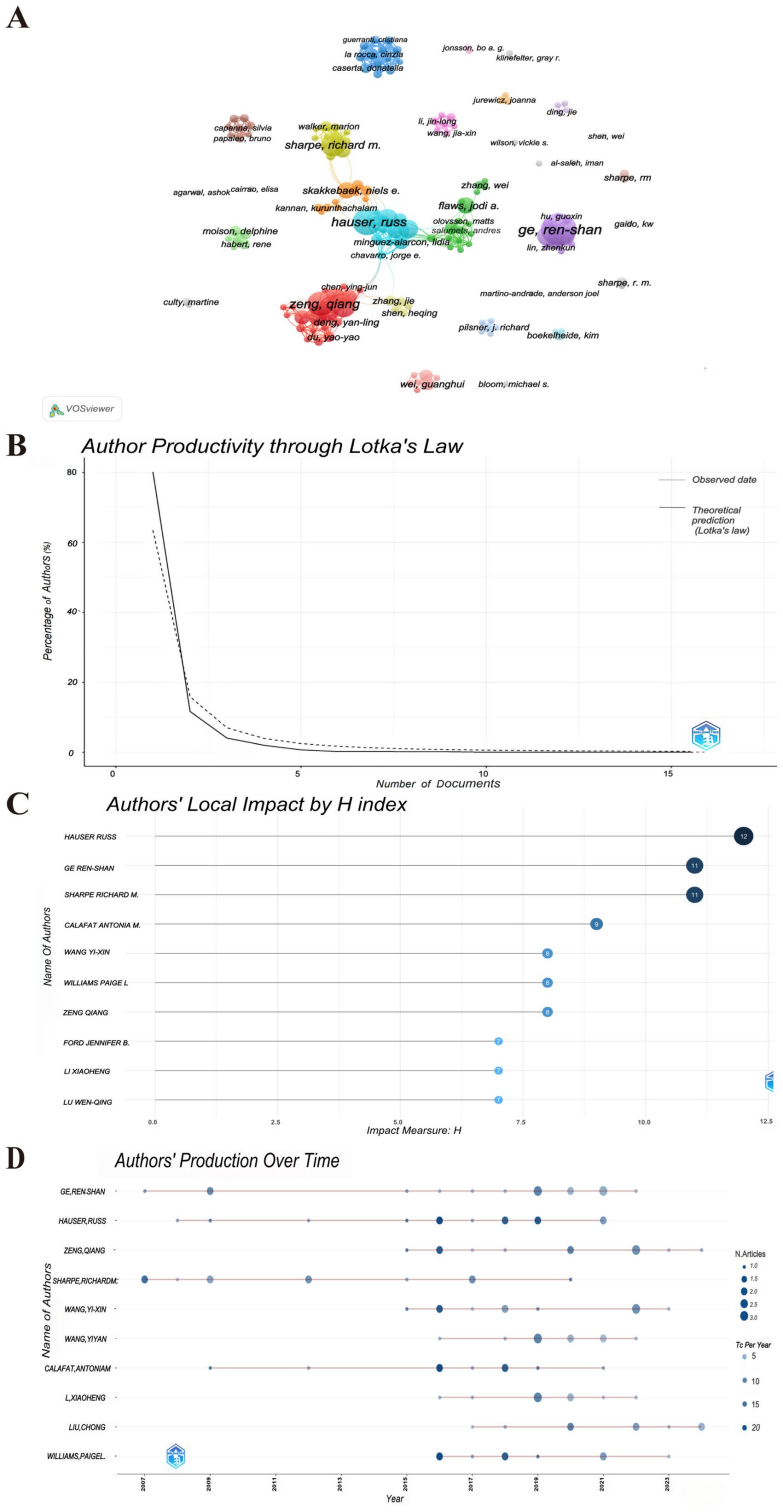
The authors contributing to publications. **(A)** Visualization cluster analysis of authors. The size of the circle nodes indicates the volume of published articles, the thickness of the connecting lines reflects the level of collaboration between authors, the colors of the nodes represent different clusters. **(B)** Author productivity through Lotka’s Law. The solid line represents the actual observed data. The dashed line represents the Lotka’s law model prediction. **(C)** Top 10 authors’ local impact by H-index. **(D)** Authors’ production over time. The color of the nodes represents the number of publications published by the author in a particular year (TC per Year), and the size of the nodes indicate the number of publications (N. Articles). The darker the bubble color, the higher the total citations per year, while lighter colors indicate lower total citations per year. The size of the bubbles is proportional to the number of articles published each year, the larger the bubble, the higher the number of publications.

Price’s Law posits that the minimum number of publications by core authors within a specific discipline can be expressed by the formula m = 0.749 * √nmax, where “nmax” denotes the number of publications attributed to the most prolific authors in that field. As indicated in Table 2, with nmax = 16, the calculation yields m ≈ 2.996. This formula identifies authors who have published three or more publications (inclusive of three) as core authors within the discipline, resulting in a total of 162 core authors. The visual analysis is presented in Figure 3A. The aggregate number of articles published by these core authors amounts to 169, which constitutes 50.6% of the overall publications. It meets the 50% criterion established by Price and is largely consistent with Lotka’s law (26, 27) (Figure 3B). Consequently, it can be inferred that the research on PAEs and infertility has developed into a relatively stable collaborative network of authors. The top 10 authors each published more than 9 papers on PAEs and infertility (Table 2). Ge RS authored the highest number of publications (*n* = 16), which received 555 citations, averaging around 35 citations per publication, and the H- index ranked second (H-index = 11). Following him is Hauser R, who has the second highest number of publications (*n* = 13), totaling 948 citations, with an average of 73 citations per publication and the highest H-index of 12 (Table 2; Figure 3C). The two authors have been engaged in interconnected research endeavors and have been disseminating academic publications since 2001 (Figure 3D).

**Table 2:** Top 10 authors contributing to publications.

Rank	Author	Publications	Citations	Mean citations per publications
1	Ge RS	16	555	35
2	Hauser R	13	948	73
3	Zeng Q	12	413	34
4	Sharpe RM	11	704	64
5	Wang YX	11	430	39
6	Wang YY	10	153	15
7	Calafat AM	9	685	76
8	Li XH	9	147	16
9	Liu C	9	125	14
10	Williams PL	9	457	51

### 3.3 Contributions of the countries and institutions

**Figure 4:**
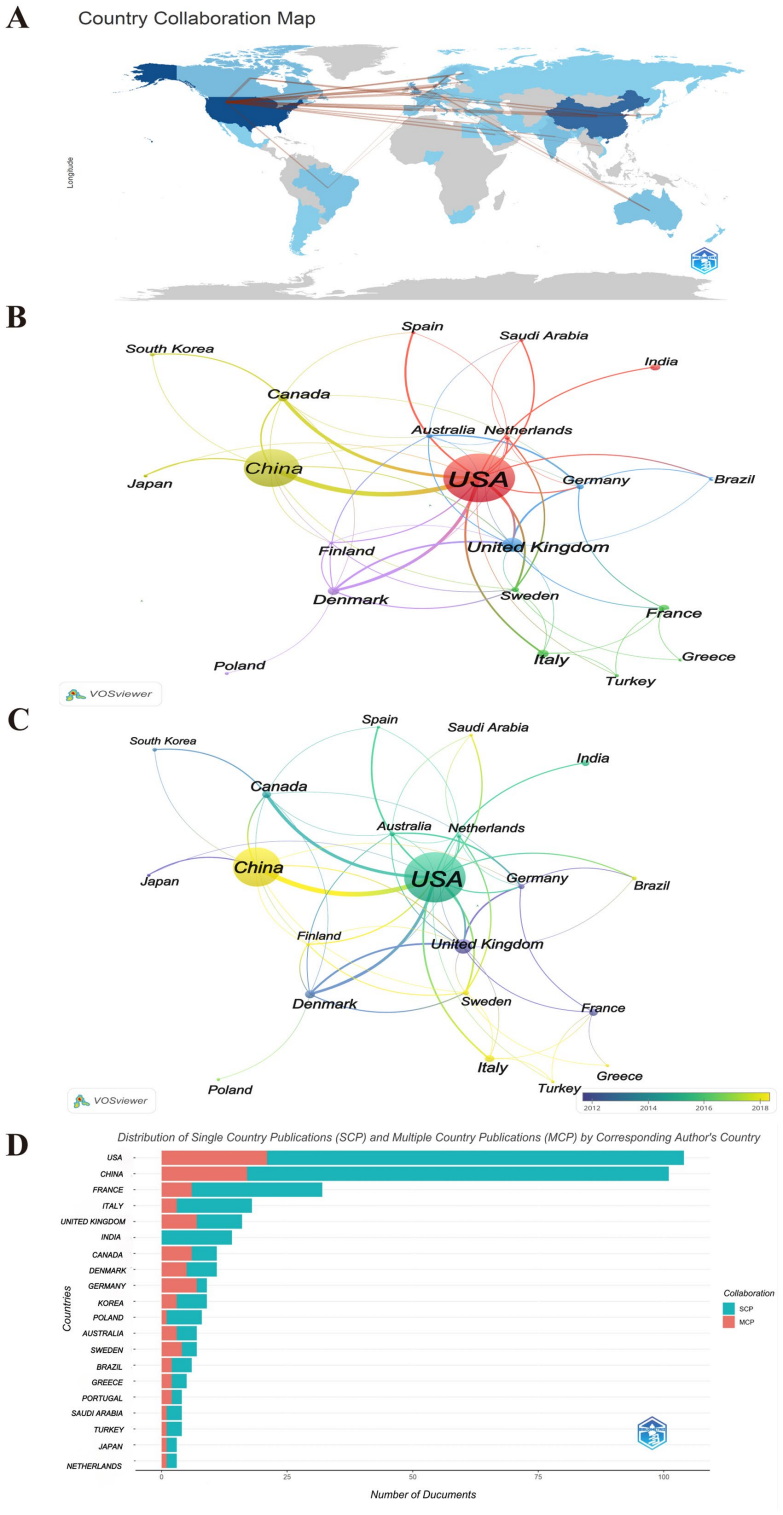
The countries contributing to publications. **(A)** Country analysis of published literatures worldwide. The thickness of the lines reflects the level of collaboration between countries, the shade of color represents the volume of publications. **(B)** Visualization cluster analysis of cooperation among countries. The size of the circle nodes indicates the volume of published articles, the thickness of the connecting lines reflects the level of collaboration between countries, the colors of the nodes represent different clusters. **(C)** Overlay visualization analysis of cooperation among countries. The color of the node represents the average year in which the keyword appears. Yellow represents the most recent research, and dark blue represents the oldest. **(D)** Distribution of Single Country Publications (SCP) and Multiple Country Publications (MCP) by Corresponding Author’s Country. MCP, multiple country publications; SCP, single country publications.

As shown in Figure 4, 21 countries have published five or more publications, revealing a significant disparity in contributions, with a significant majority of the publications authored by researchers from China and the USA (Figures 4A,D). Table 3 lists the top 10 countries in this research area. It shows that American researchers have produced the highest number of papers, with 136 publications, which constitutes 33.5% of the total in this field. These works have garnered 9,401 citations, averaging 69 citations per publication. China ranks second with 106 publications, accounting for 26.11% of the total publications, receiving 3,009 citations, only averaging 28 citations per publication, indicating a need for enhancement in the quality of research. Denmark stands out for having the most mean citations per publication (mean citations = 154), with 22 articles receiving a total of 3,378 citations.

**Table 3:** Top 10 countries contributing to publications.

Rank	Country	Publications	Publications (%)	Citations	Mean citations per publication
1	USA	136	33.50%	9,401	69
2	China	106	26.11%	3,009	28
3	United Kingdom	39	9.61%	3,367	86
4	Denmark	22	5.42%	3,378	154
5	Italy	21	5.17%	712	34
6	France	20	4.93%	986	49
7	Canada	20	4.93%	1,090	55
8	India	17	4.19%	933	55
9	Sweden	14	3.45%	350	25
10	Germany	14	3.45%	1,369	98

As shown in Figures 4A,B, obviously, there is evidence of increased collaboration among the USA, China, Denmark, United Kingdom. Regarding the concentration time of publications, the USA issued publications approximately in 2016, China in 2018, the United Kingdom in 2011, and Denmark around 2012 (Figure 4C). It also highlights the significant leading positions of the USA and China in this research field, which may reflect their important research capabilities and international influence in related fields (Figure 4D). Simultaneously, this reveals the varying cooperation patterns of publications in this field among nations. The majority of countries, including China and the USA, predominantly engage in Single Country Publications (SCP), In contrast, a limited number of countries, such as Canada, Germany, and Sweden, demonstrate a higher frequency of multi-country publications (MCP). This indicates that research within this domain is predominantly conducted by individual institutions or universities, resulting in a scarcity of multi-center collaborations. While this situation may impede international cooperation and the exchange of knowledge, it simultaneously presents opportunities to strengthen global scientific collaboration.

Table 4 presents the top 10 institutions, mostly located in the USA and China. Huazhong University of Science and Technology in China has the highest publication output (*n* = 20). The top 10 institutions in terms of publications volume such as Wenzhou Medical University, Nanjing Medical University and Shanghai Jiao Tong University are also all located in China. Center for Disease Control and Prevention, located in the USA, records the greatest number of citations (citations = 2,331) and the highest mean citations per publication (mean citations = 194). The institutions with the second highest number of publications (Harvard T. H. Chan School of Public Health) and mean citations per publications (Massachusetts General Hospital) are both located in the USA. The data suggests that researchers from the USA and China have significantly contributed to the research on PAEs and infertility.

**Table 4:** Top 10 institutions contributing to publications.

Institutions	Publications	Citations	Mean citations per publication	Countries
Huazhong University of Science and Technology	20	553	28	China
Harvard T. H. Chan School of Public Health	18	536	30	USA
University of Edinburgh	17	1,502	88	United Kingdom
Wenzhou Medical University	14	287	21	China
Nanjing Medical University	13	183	14	China
Center for Disease Control and Prevention	12	2,331	194	China
Massachusetts General Hospital	11	1,011	92	USA
University of Illinois	11	944	86	USA
Brown University	11	560	51	USA
Shanghai Jiao Tong University	10	71	7	China

### 3.4 Analysis of the journals and co-cited journals

**Figure 5:**
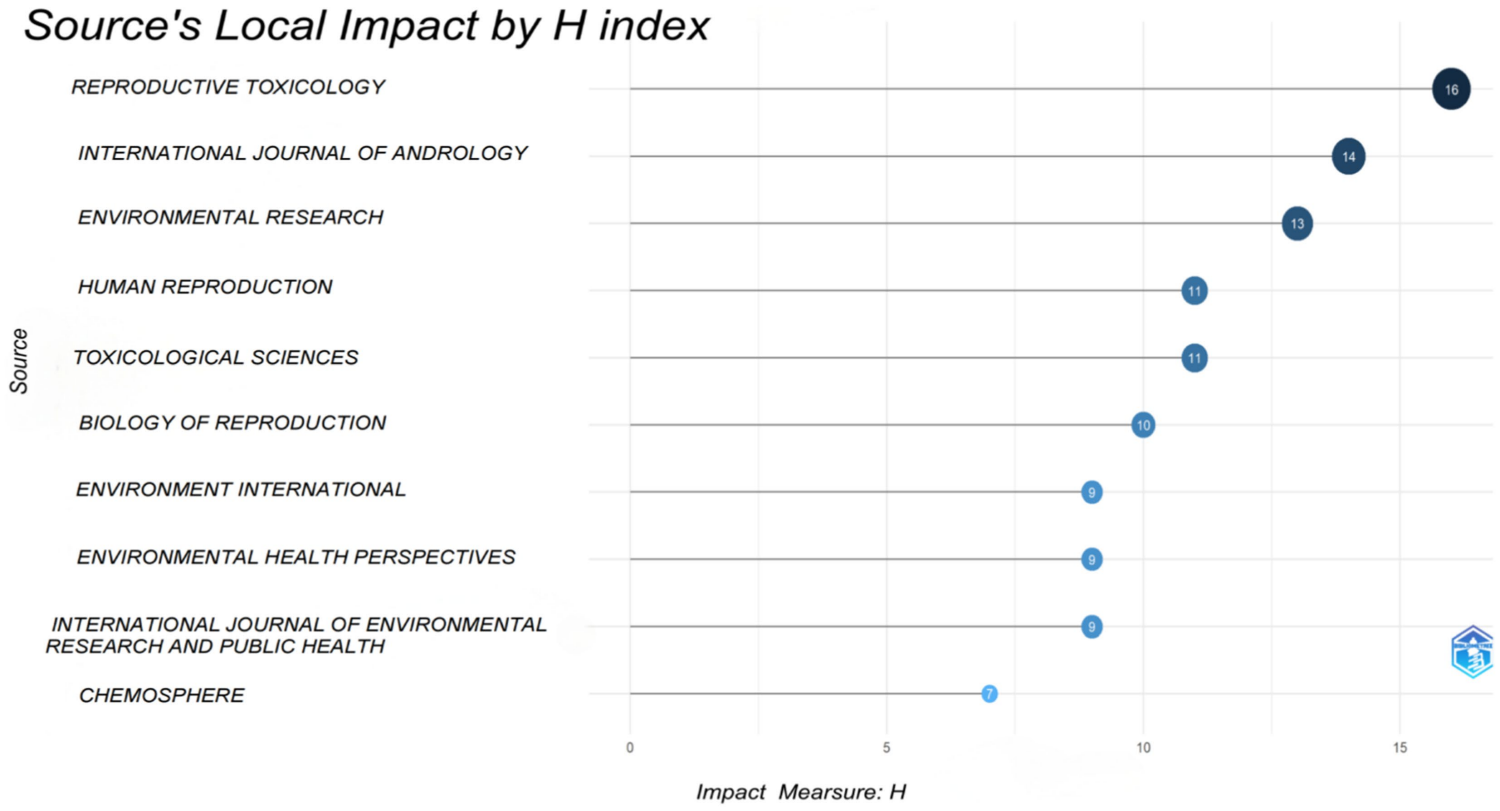
Journals’ local impact by H-index.

As indicated in Table 5, Reproductive Toxicology (20 publications, IF: 3.3, JCR: Q2) has published the highest number of articles concerning PAEs and infertility. Following this, the other four journals in the top five are Environment International (15 publications, IF: 10.3, JCR: Q1), Environmental Research (14 publications, IF: 7.7, JCR: Q1), and the International Journal of Andrology (14 publications, IF: 3.695, JCR: Q2), Toxicological Sciences (13 publications, IF: 3.4, JCR: Q2). Collectively, the top 10 journals account for approximately 32.2% of the total publications (131 out of 406). Notably, only Environment International, Environmental Research, Human Reproduction and Environmental Health Perspectives possess impact factors exceeding 5.0 and the H-index ranks in the top ten (Figure 5), suggesting that there is a need for enhancement in the publishing standards of researchers in this field.

**Table 5:** Top 10 core journals contributing to publications.

Rank	Journals	Publications	Citations	Mean citations per publication	IF (2024)	JCR
1	Reproductive Toxicology	20	1,037	52	3.3	Q2
2	Environment International	15	566	38	10.3	Q1
3	Environmental Research	14	684	49	7.7	Q1
4	International Journal of Andrology	14	1704	122	3.695	Q2
5	Toxicological Sciences	13	873	67	3.4	Q2
6	Frontiers In Endocrinology	12	1,241	103	3.9	Q1
7	Human Reproduction	12	400	33	6.0	Q1
8	Environmental Pollution	11	194	18	7.6	Q1
9	Biology of Reproduction	10	2,275	228	3.1	Q1
10	Environmental Health Perspectives	10	573	57	10.1	Q1

**Figure 6:**
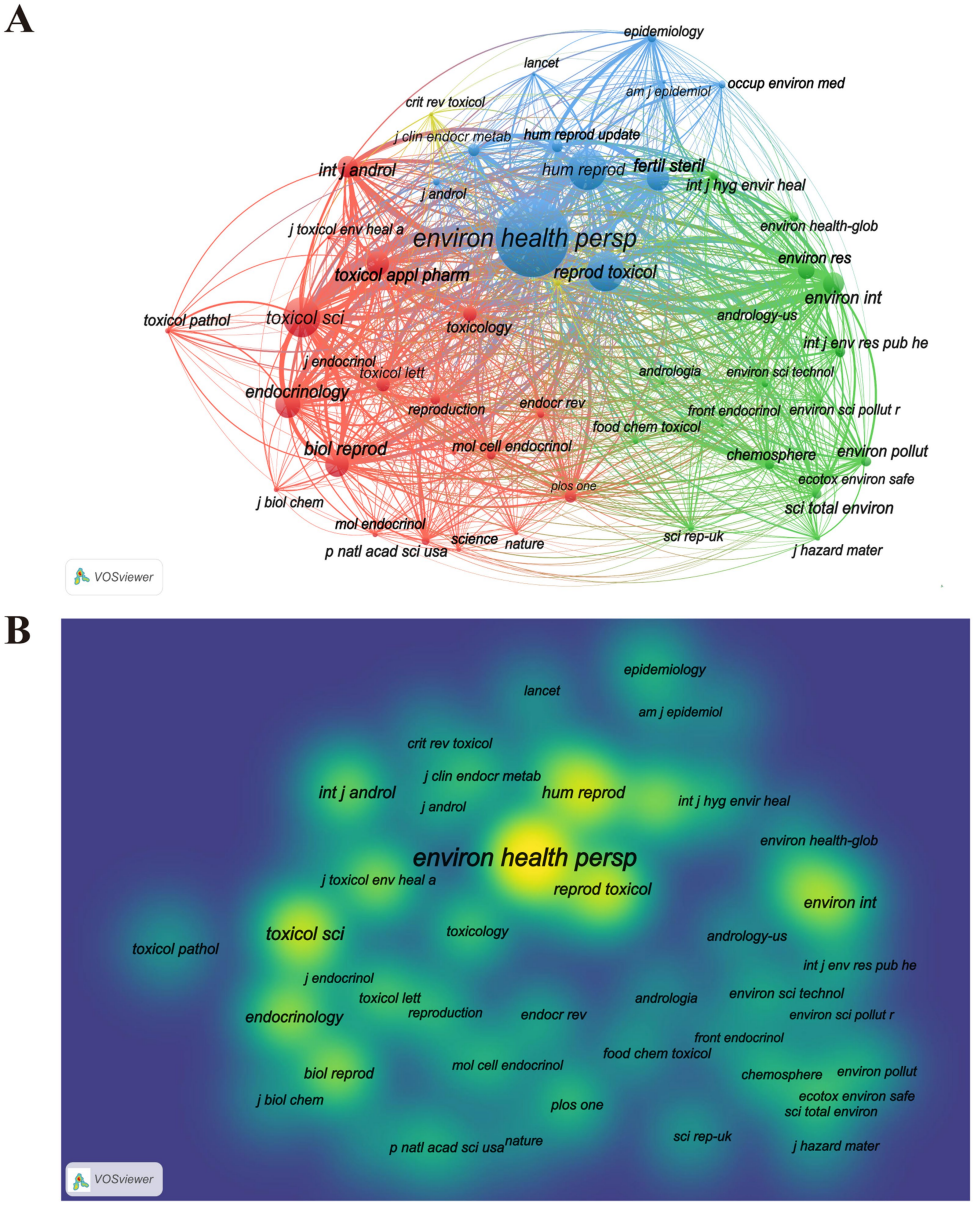
Co-citation analysis of cited journals. **(A)** Visualization network of co-cited journals. The size of the circle nodes indicates the volume of published articles, the thickness of the connecting lines reflects the level of collaboration between countries, the colors of the nodes represent different clusters. **(B)** Visualization density analysis of co-cited journals. The VOS clustering method is adopted. The each circle corresponds to each journal. The yellower areas indicate a higher volume of publications, the greener areas indicate less publications. Brighter colors correspond to more concentrated nodes and stronger correlations, while darker colors correspond to more dispersed nodes and weaker correlations.

As shown in Figure 6, the visualization analysis of 50 journals that have received over 100 citations reveals a co-citation network comprising four distinct clusters, each represented by a different color in the figure. The four clusters can be characterized as follows in Figure 6A. The red cluster includes journals are mainly in the fields of reproductive biology and toxicology. The main purpose of citing these journals is to analyze the influence of PAEs on reproductive endocrinology and biology, thereby providing both theoretical and empirical support for related research endeavors. The green cluster includes journals within the environmental science domain, emphasizing ecotoxicology and environmental safety. This cluster addresses the effects of environmental pollutants on living organisms and ecosystems, as well as risk assessment and management, thus highlighting the ecological implications of pollution. The blue cluster primarily pertains to reproductive endocrinology and epidemiology, offering epidemiological data and theoretical frameworks to support research in these areas. The smallest yellow cluster is related to the field of toxicology.

The three most frequently co-cited journals within this network are Environmental Health Perspectives (2,174 citations, IF = 10.1, JCR: Q1), Human Reproduction (1,148 citations, IF = 6, JCR: Q1), and Toxicological Sciences (1,107 citations, IF = 3.4, JCR: Q1), all of which are recognized as high-quality journals in the Journal Citation Reports (Figure 6B; Table 6).

**Table 6:** Top 10 co-cited journals contributing to publications.

Rank	Journals	Citations	IF	JCR
1	Environmental Health Perspectives	2,174	10.1	Q1
2	Human Reproduction	1,148	6.1	Q1
3	Toxicological Sciences	1,107	3.4	Q1
4	Reproductive Toxicology	1,105	3.3	Q2
5	Endocrinology	746	3.51	Q1
6	Biology of Reproduction	709	3.1	Q1
7	Toxicology and Applied Pharmacology	671	3.42	Q2
8	Fertility and Sterility	665	6.7	Q1
9	Environment International	629	10.3	Q1
10	International Journal of Andrology	587	3.695	Q2

### 3.5 Analysis of co-cited references

**Figure 7:**
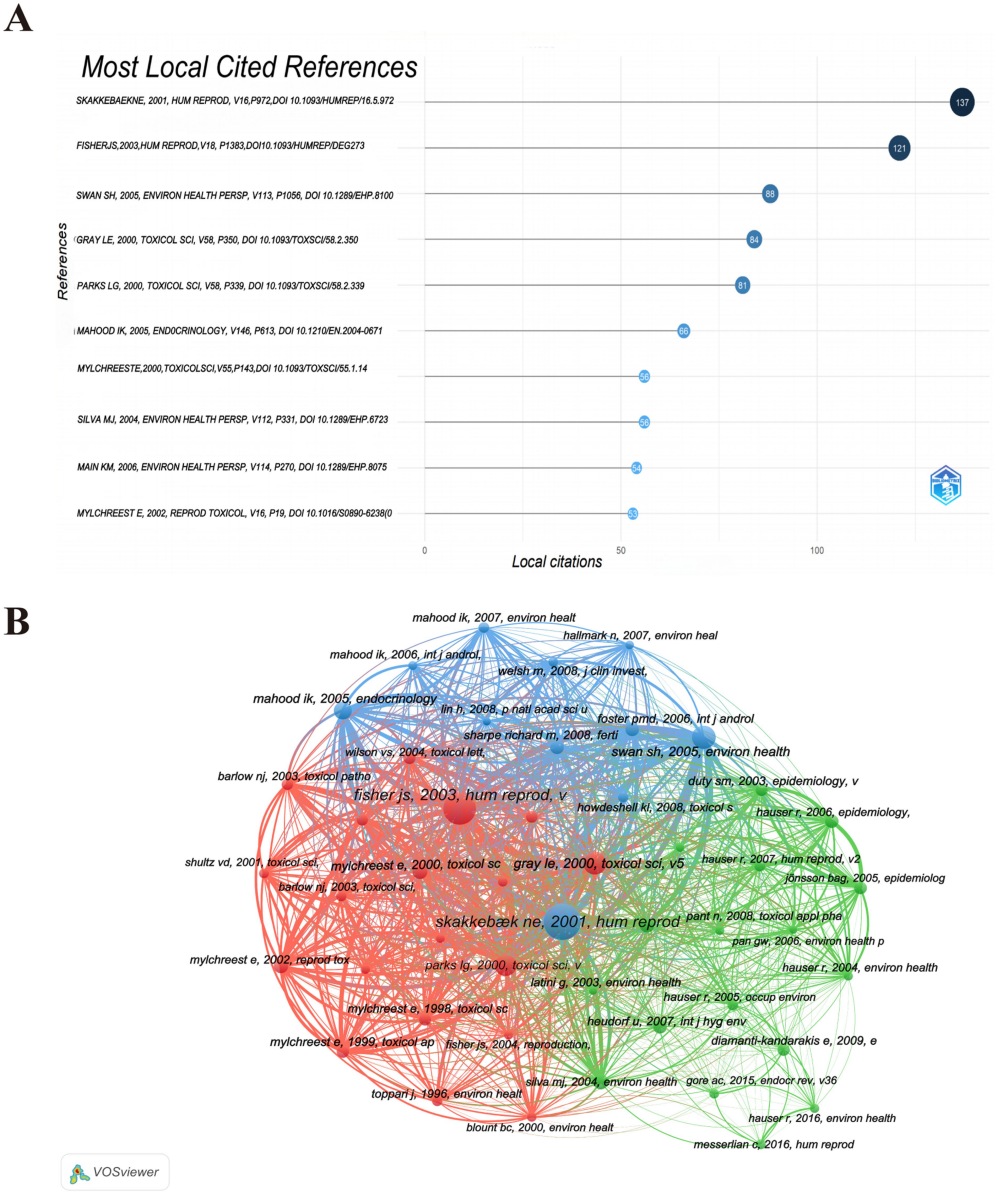
Co-citation analysis of cited references. **(A)** Top 10 co-cited references. **(B)** Visualization network of co-cited references. The size of the circle nodes indicates the volume of citations, the thickness of the connecting lines reflects the level of collaboration, the colors of the nodes represent different clusters.

In addition, the co-cited references have been subjected to analysis. As illustrated in Figure 7A and Supplementary Table 1, within the top 10 most co-cited references in the PAEs and infertility from 2001 to 2024.

Subsequently, a co-citation map of the cited references was generated using VOSviewer, with a threshold set for the minimum number of citations for a cited document at 30. This criterion resulted in a selection of 47 documents for the co-citation analysis. The co-citation relationship map, as depicted in Figure 7B, reveals that the co-citation network of frequently cited references can be categorized into three distinct clusters, each represented by a different color. The red cluster predominantly encompasses research literature pertaining to reproduction, environmental studies, and toxicology, whereas the blue and green clusters are more focused on environmental research. Notably, the majority of the highly co-cited references were published between 2000 and 2008. Through an examination of pertinent policies and literature, alongside an analysis of the research characteristics and historical context within the field of environmental health, we ascertain that this phenomenon is predominantly influenced by a confluence of factors: shifts in national policy, transitions in research focus, advancements in technology, and the Matthew Effect associated with seminal literature. Several key points emerge from this analysis:

- The period from 2000 to 2008 is recognized as a “golden decade” for research on PAEs reproductive toxicity, marked by significant mechanistic discoveries and human evidence. Foundational studies established critical theoretical frameworks: Skakkebaek (28) introduced the concept of Testicular Dysgenesis Syndrome (TDS) to unify various male reproductive disorders; Gray (29) illustrated the estrogenic disruption of steroidogenesis; and Swan et al. (30) confirmed the prenatal toxicity of phthalates through the observation of reduced anogenital distance (AGD) in humans. Concurrently, regulatory initiatives such as the EU REACH (2005) (31), the US Consumer Product Safety Improvement Act (CPSIA) (2008) (32), and China’s GB 24613-2009 (33) imposed restrictions on PAEs in consumer products, further fueled by public discourse surrounding the “global sperm decline” and “infertility” (34, 35). These studies became highly co-cited, establishing enduring nodes within the research network.
- Following 2008, a consensus on core mechanisms redirected research towards specialized applications and technological innovations. New areas of focus included safety assessments of alternative substances (e.g., DINCH), transgenerational epigenetics (36, 37), and mixture toxicology. The adoption of advanced methodologies—such as multi-omics integrationand high-throughput exposure assessment—facilitated detailed analyzes but resulted in fragmented citations across numerous studies, hindering the formation of new consensus (38–40).
- The continued prominence of literature published prior to 2008 exemplifies the Matthew Effect (41), wherein foundational papers gain reinforcement through preferential citation. Highly cited works are more readily prioritized in searches and referenced by subsequent researchers, engendering a “rich-get-richer” dynamic. Contemporary advancements now necessitate disproportionate resources—such as multi-center cohorts, global data integration, or mechanistic-clinical translation (42, 43)—resulting in elevated thresholds for impact. While future co-citation networks may coalesce around themes such as epigenetic regulation, multi-pollutant interactions, or studies on exposure disparities, the theoretical framework established during the 2000–2008 period remains a foundational anchor for the field, illustrating the path dependency inherent in environmental health research. Although subsequent investigations have expanded upon these mechanisms, the core theoretical framework continues to be rooted in the pivotal discoveries of that era. Therefore, the co-citation at this stage is more prominently concentrated.

### 3.6 Analysis of keywords

**Figure 8:**
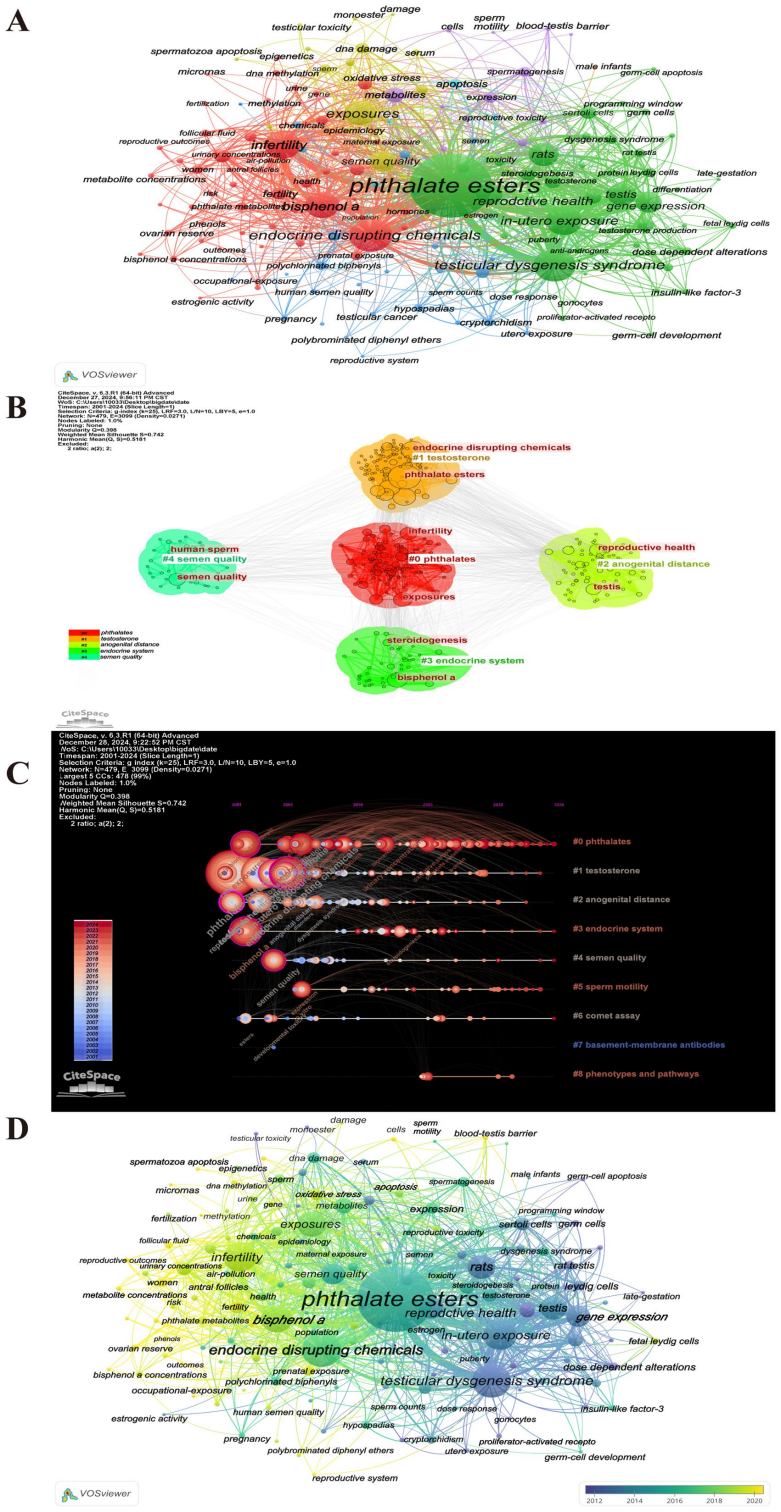
Visual analysis of keywords. **(A)** Clustering analysis of keywords. **(B)** Top 5 clustering network map of keywords. The network diagram is plotted using LSI clustering algorithm. Different colors to represent different categories, each node represents a keyword, and the edges indicate co-occurrence similarity. **(C)** Timeline zone analysis of keyword clustering. The same horizontal line represent the same clusters, and the timeline can show how these clusters evolve over time. The bluer the color, the earlier it appears, and the redder the color, the later it appears. **(D)** Overlay visualization analysis of cooperation among keywords. The color of node represents the average year in which the keyword appears. Yellow represents the most recent research, and dark blue represents the oldest.

**Figure 9:**
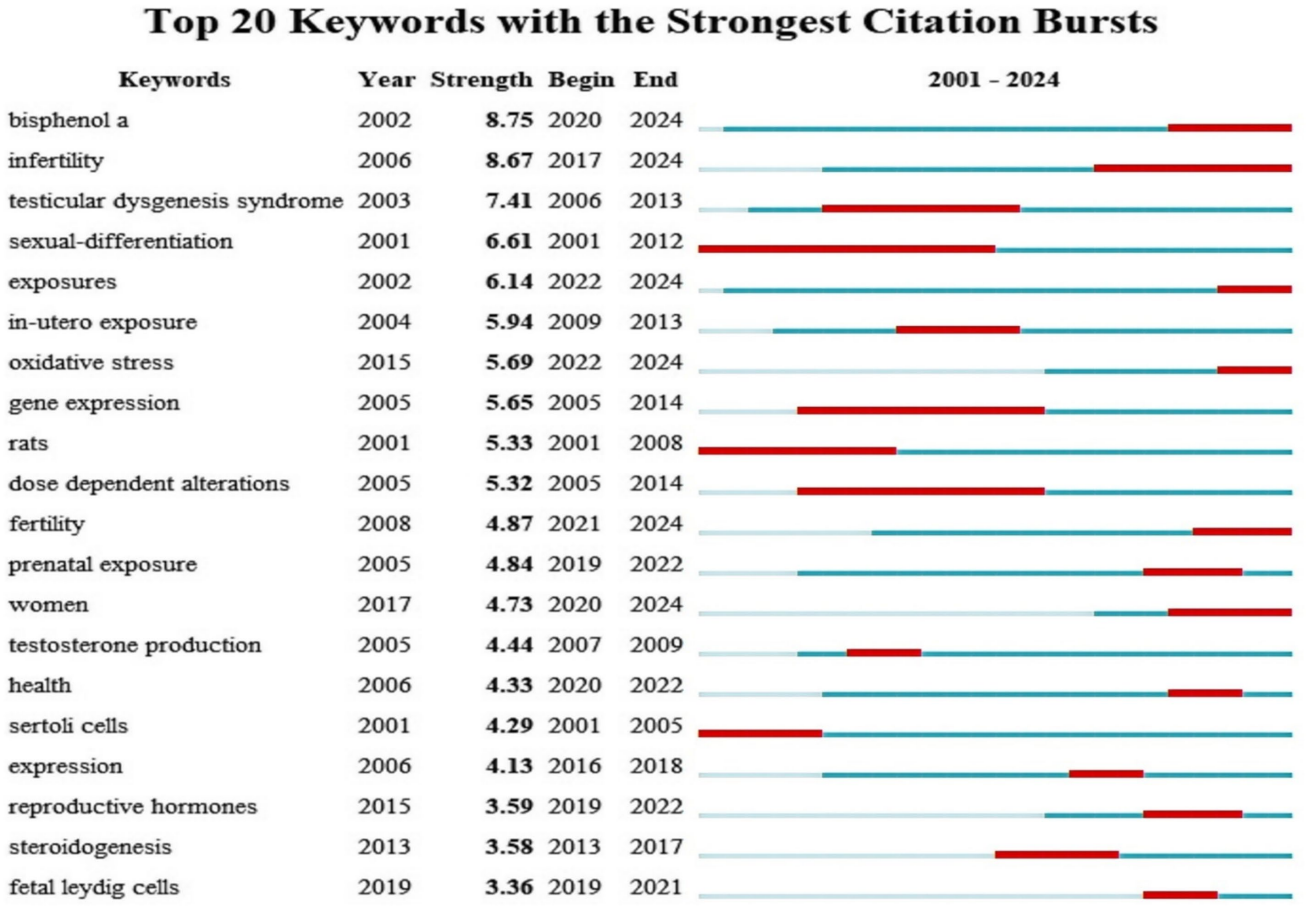
Top 20 representative keywords with the strongest citation bursts. The red bar represents the duration of keywords burst, “strength” represents the burst intensity, “Year” indicates the calendar year when a keyword demonstrated a significant citation burst. “Begin” indicates the initial year of a citation surge. “End” marks the terminal year when scholarly attention to the keyword began to decline.

To ascertain the predominant research hotspots in the domains of PAEs and infertility, as well as to analyze the temporal trends in keyword usage, a keyword co-occurrence visualization analysis was conducted using VOSviewer and Citespace (Figure 8). After collecting a total of 1,651 keywords, a focused analysis of keyword co-occurrences was performed on a subset of 161 keywords that appeared more than five times (Figure 8A). In the “network visualization” depicted in Figure 8A, each node represents a keyword, with its size corresponding to its frequency of occurrence. The clustering results indicated that “phthalates” and “testosterone” are the two most significant areas and hotspots of research related to PAEs and infertility, and the “endocrine system” also remains a persistent hotspot (Figures 8B,C). The colors in the “overlay visualization” presented in Figure 8D reflect the most common publication year for each keyword, revealing that the majority of keywords associated with PAEs and infertility were published since 2016. Figure 9 highlights top 20 burst keywords, which exemplifies the phenomenon of certain keywords emerging with increased frequency during specific time periods, thereby indicating recent and future research trends. The top three keywords for the burst intensity are “bisphenol a,” “infertility” and “testicular dysgenesis syndrome.” Notably, after 2013, the focus on “testicular dysgenesis syndrome” has diminished. In contrast, the terms “infertility,” “bisphenol a,” “women” and “oxidative stress” have attracted considerable academic attention since 2017 and are anticipated to continue being central themes in forthcoming research.

Additionally, our analysis of outbreak keywords indicates that the topics of PAEs, “infertility,” and “fertility” have consistently attracted attention over the past two decades, with a marked increase in interest in recent years. So we can conclude that the research focus on PAEs and fertility has evolved through three distinct phases: the initial dominance of environmental exposure pathways (2001–2008), the rise and transformative influence of life exposure pathways (2009–2018), and the recent prominence of multiple pathways (2019–2024), as detailed below.

- The initial phase (2001–2008) was characterized by a focus on environmental exposure pathways.
  The keyword “exposures” emerged in 2002 with an intensity of 6.14 and has persisted until 2024. Similarly, “bisphenol A (BPA)” appeared in 2002 with an intensity of 8.75, also continuing through 2024. This indicates that early research was directed towards various exposure pathways, with BPA, a prevalent environmental chemical, receiving considerable attention regarding its effects on reproductive health. Furthermore, the keyword “rats” experienced a surge in prominence during 2001–2008, suggesting that the early research phase was dominated by investigations into the reproductive toxicity mechanisms of environmental pollutants using animal models, with a focus on underlying pathological endpoints such as testicular dysplasia.
- Intermediate stage (2009–2018): notable influence of life exposure pathways.
  The term “in-utero exposure” has been around since 2004 with an intensity of 5.94 and peaked between 2009 and 2013. This trend underscores the increasing apprehension regarding the reproductive health implications of chemicals found in common consumer and personal care products. Such developments emphasize the significance of life exposure pathways during this medium-term period.
- Near-term phase (2019–2024): emergence of multiple exposure pathways.
  The term “exposures” has been utilized since 2002, with an intensity of 6.14, and its prominence in research is projected to persist through 2024, particularly from 2022 to 2024. Concurrently, “prenatal exposure,” which has been studied since 2005, garnered significant attention primarily from 2019 to 2022, although interest has waned in recent times. These shifts suggest a gradual diversification in the investigation of exposure pathways, transitioning from a focus on early intrauterine exposure to encompass a wider array of exposures throughout pregnancy, postnatal development, and into adulthood. This trend reflects a comprehensive scholarly emphasis on the effects of exposure across various life stages. Notably, prenatal exposure is emerging as a central focus of research, facilitating advancements in dose-dependent modifications and toxicity assessments of alternatives, such as the evaluation of BPA. In conclusion, the research on exposure pathways shows a trend of diversification and in-depth, and it is necessary to further grasp the mechanism of complex exposure factors of PAEs on infertility in future research.

Meanwhile, since 2022, the burst strength of the keyword “exposure” in PAEs research has significantly increased, becoming the core hub connecting “PAEs” and “infertility.” The academic community is no longer satisfied with the “detected concentration” itself but has shifted its focus to “exposure characteristics of different countries and populations” and “exposure dose–response relationships.” Through multi-dimensional analysis, this study reveals the following potential causes:

- Exposure characteristics of different countries and populations.
  Geographical and socioeconomic gradients, when analyzed through regulatory and cultural perspectives, elucidate the differences in exposure profiles among countries. This framework delineates a comprehensive causal pathway linking exposure to vulnerability and subsequent health outcomes. Consequently, the application of stratification at the national level not only clarifies the reasons behind the heightened susceptibility of specific groups to reproductive toxicity but also offers empirical support for the formulation of targeted policy interventions.
  1. The Unique Value of Nations as “Composite Exposure Units.”
    The same chemical may exhibit vastly different exposure patterns across countries due to varying regulatory thresholds and cultural practices. The USA imposes stricter limits on DEHP (6 μg/L) compared to China and WHO standards (8 μg/L), while China has further implemented controls for DBP (3 μg/L) and DEP (300 μg/L) (44, 45). The European Union has developed health guidance values for urinary metabolites (e.g., DEHP metabolite limit of 0.5 mg/L) by banning traditional PAEs and promoting alternatives (DINCH/DEHTP) (46). This policy divergence drives the intercontinental divergence of exposure trends. For instance, Global human samples (mainly urine) showed that the metabolites of DEHP were the highest in Asia and mEP was the highest in America (47); meanwhile, PAEs exposure in Asia showed an increasing trend, while PAEs exposure in Europe and America showed a decreasing trend, and the exposure to alternatives increased (48). The metabolites of DEHP (5oxo-MEHP, 5OH-MEHP) and DnBP (MnBP) in China continued to rise, and the average level of MnBP detected in the national sample of South Korea was as high as 41.7 μg/g, with the exposure levels of women and the elderly being particularly (48, 49). The metabolism of DEHP decreased significantly in North America, while the replacement DEHT/DINCH continued to increase; the detection rate of DINCH metabolites in young German urine increased from 6.7% in 2006 to 100% in 2017, and the median concentration increased by 367% in 5 years (48, 50).
  2. The “Exposure Inversion” Phenomenon in Socioeconomic Hierarchy.
    Exposure patterns exhibit complex socioeconomic gradients with an inverted distribution pattern. High-risk groups follow the inverse relationship with national development levels. In high-income countries, low-income communities often face higher chemical exposure due to living in polluted industrial zones or consuming substandard food products. Conversely, in some middle-and low-income countries, affluent populations may become high-exposure groups through imported cosmetics, e-waste dismantling industries, or occupational solvent use (51). Epidemiological studies have shown that the levels of low molecular weight (∑LMW) metabolites in the US population with low socioeconomic status, non-white ethnicity and foreign-born individuals are 11–65% higher than those in the white population (52). For every 10% increase in energy supply of ultra-processed food for pregnant women, urine ΣDEHP increased by 13.1%, and the increase of urine ΣDEHP was further aggravated by low income and education status by 1.9 and 1.4% (53). And, in low-income regions of the world (such as Middle East-South Asia, East Asia-Pacific), DBP exposure increased exponentially, and the trend was aggravated by heavy metals such as PAEs released by open burning of plastic waste, BPA, PAHs and other pollutants (54, 55).
  3. Geographical Differentiation and Spatial Polarization.
    The exposure patterns of PAEs and their impacts on infertility demonstrate significant geographical variations. Studies reveal spatial polarization in water and soil contamination: global water PAEs pollution is most severe in Nigeria and least in China. The average concentration of DBP/DEP exceeds 15,573 mg/L. In China’s facility agriculture sector, soils in southwestern regions and South Carolina exhibit the highest total PAE levels (0.053–5.663 mg·kg^−1^), with DEHP + DnBP accounting for over 70% of total concentrations (56). The Paris region exhibits a geographical pattern of PAEs/BPA pollution characterized by “urban core concentration, secondary agricultural impact, and forest marginalization”: After applying sludge to urban soil, DEHP levels surged eightfold while BPA increased threefold. Notably, the measured DEHP concentration (0.16 μg/g) fell below the predicted threshold (0.3 μg/g), indicating strong degradation or fixation in densely populated urban areas. Vertically, low-molecular-weight PAEs showed a positive gradient with soil depth, whereas high-molecular-weight PAEs exhibited a negative gradient (57).
- Exposure dose–response relationship.
  The limited number of dose–response studies before 2022 has hindered further interpretation of causality (58). After 2022, the critical role of exposure-dose–response relationships in PAE-induced infertility became widely recognized. Yiwei Fang (59) conducted a dose-effect metabolomics analysis on HTR-8/Svneo cells exposed to MEHP. The study revealed that ≥5 μM MEHP significantly disrupted 300 metabolic profiles in these cells, with 5’-UMP (E_−10_ = 0.1 μM) and N-acetylcoramine (E_+10_ = 0.11 μM) identified as biomarkers. These findings provide crucial evidence for developing more refined dose-effect models and risk assessments.

## 4 Discussion

The bibliometric analysis serves as a valuable tool for researchers to systematically analyze and evaluate the publications, thereby elucidating core themes and emerging trends in the specific field (60). In this context, we successfully summarized the research general situation, developmental trajectories, research hotspots and frontiers concerning PAEs and infertility, by examining various aspects such as the numbers of publications, the co-occurrence visual analysis of keywords and the contributions from authors, institutions, countries, journals.

### 4.1 The general situation

Regarding the global research trends related to PAEs and infertility from 2001 to 2024, there have been approximately 406 publications, with an average of about 17 publications per year. This indicates a significant potential for further exploration and investigation within this area. The overall trend in publication numbers has shown a consistent increase, particularly notable in the articles published since 2015, which exceeds the average annual output. This trend suggests a growing interest and attention towards research in this domain in recent years.

Furthermore, we conducted an analysis of the authors, countries, institutions, and journals that have made the most substantial contributions to the publications on PAEs and infertility. Reproductive Toxicology, Environment International, International Journal of Andrology, Human Reproduction, Environmental Health Perspectives, Biology of Reproduction, the number of published articles and cited times of these journals are also in the top 10, involving fields such as environmental science, public health, environmental hygiene, and reproductive health. This indicates that the theme of PAEs and infertility has a wide range of coverage and strong influence. Ge RS, Hauser R, Zeng Q, Sharpe RM and Wang YX are the top 10 ranked authors in terms of published articles and with the top 5 H-index. This indicates that these individuals are recognized as leading figures in this field of research, their publications have great reference value for the researchers. And more than half of the publications are written by researchers from China and the USA. The top 10 institutions in terms of publications volume such as Huazhong University of Science and Technology, Wenzhou Medical University, Nanjing Medical University and Shanghai Jiao Tong University are also all located in China. However, the average citation per publication of these institutions is not ideal, indicating that their publication quality still needs further improvement. Center for Disease Control and Prevention in the USA, Harvard T. H. Chan School of Public Health, Massachusetts General Hospital, University of Illinois and Brown University are all the top 10 institutions to the contributions in the USA, especially the quality of literature published is far ahead. This shows that the USA and China are the countries that have made the greatest contributions in this field. Other countries, such as Denmark and United Kingdom, also cooperate closely with authors and institutions in China and the USA, and jointly promote the development of this research. This demonstrates the crucial importance of international cooperation in academic research and innovation. The data indicates that SCPs are predominant in the field of PAEs-infertility research; however, they are deficient in comprehensive cross-national studies. SCPs serve as indicators of a nation’s autonomous research capabilities, yet their findings may not be broadly applicable. Conversely, MCPs embody international collaboration, which can enrich research perspectives and amplify impact. Nonetheless, MCPs encounter difficulties related to coordination and data management. To further the advancement of PAEs-infertility research, it is imperative for countries to adopt appropriate collaboration models, bolster multi-center partnerships, establish effective cooperative frameworks, and facilitate knowledge sharing to enhance global scientific collaboration.

### 4.2 Hotspots and frontiers

According to keyword clustering and outbreak words, it is obvious that “bisphenol a,” “exposures” and “testicular dysgenesis syndrome” have high outbreak intensity, which has been the focus of research, while “oxidative stress” and “women” “infertility” are new research hotspots about PAEs in recent years. Through an analysis of these keywords and related cited references, we have developed a more nuanced understanding of research priorities and frontiers of its etiology, pathogenesis, diagnosis, treatment.

#### 4.2.1 Etiology and pathogenesis

N. E. Skakkebaek (28) has highlighted that the contamination by persistent endocrine disruptors represents a significant global issue concerning human health. It is imperative to examine the deterioration of human reproductive health attributed to detrimental environmental disrupting chemicals. PAEs mainly affect human reproductive health through in-uterine exposure, prenatal exposure and other forms of exposure, works with bisphenol a (BPA) and other endocrine disrupting chemicals to cause infertility (61). Studies show that BPA and PAEs are both endocrine disruptors, with chemical structures similar to steroid hormones. They can interact with estrogen receptors (ER) and other components, disrupting the normal function of the endocrine system, hindering the development of germ cells, and subsequently affecting the reproductive system (62). Additionally, research indicates that BPA can also interfere with the normal function of the HPG axis by inhibiting the synthesis of neuropeptide B, blocking the release of GnRH from the hypothalamus, and thereby affecting the secretion of pituitary gonadotropins, leading to ovarian dysfunction and impacting ovulation and follicle development (63). In real-world environments, people are often exposed to multiple endocrine disruptors simultaneously, and BPA and PAEs may have synergistic effects, collectively impacting reproductive health. Therefore, the role of mixed etiology should be the emphasis of subsequent investigation. Oxidative stress represents a mechanism that merits additional investigation in future research endeavors in PAEs and infertility. Numerous in vivoanimal studies and human epidemiological investigations have found that PAEs can act as inducers of reactive oxygen species (ROS) (64–72), inhibit the expression of antioxidant superoxide dismutase 1 (SOD1) (73). Experimental research shows PAEs can activate the PI3K/AKT and NF-κB signaling pathways to damage gene expression, mitochondrial apoptosis and cell death caused by oxidative stress, resulting in neurotoxicity, reproductive toxicity and even genotoxicity (74–77). Neurotoxicity refers to the increase in hippocampal weight and neuron count caused by oxidative stress in the hippocampus, which adversely regulates the functions in rats (45, 46). This also suggests potential disruption of human nervous systems (78, 79). Reproductive toxicity refers that PAEs can not only have the ability to bind to hormone receptors but also disrupt the hypothalamic–pituitary-gonadal (HPG) axis due to oxidative stress (80). This exposure is associated with leading to a decrease in reproductive hormones such as luteinizing hormone (LH), follicle-stimulating hormone (FSH), estradiol (E2), and testosterone (T) in humans (81). Research in male rats also indicates that damage to male reproductive health primarily encompasses reproductive toxicity, which includes testicular oxidative stress, testicular weight loss, degeneration of germ cells (leying cells, sertoli cells), and abnormal sperm, collectively referred to as TDS (60, 82). While historical research emphasized male reproductive health, recent animal studies indicates that the risk of female exposure is actually higher. Studies in female mice show ovarian oxidative stress leads to mitochondrial dysfunction and DNA damage, inhibiting the growth of sinus follicles and inducing oocyte fragmentation (83). This significantly harms the ovarian function, resulting in hormonal imbalances and accelerating the onset of endometriosis, reproductive aging, infertility, elevated abortion rates, and pregnancy complications (84, 85). Animal studies further indicate that preconception and prenatal exposure to PAEs not only affects the individuals throughout life but also has a serious impact on the reproductive health of offspring, which is referred to as the transgenerational effect (86, 87). This is associated with hormone secretion, oxidative stress, which may lead to metabolic disorders, nerve damage, and reproductive disorders in the fetus by altering the endocrine system (87). Collectively, these PAE-induced effects across sexes and generations can contribute to infertility. Be undisputed, the infertility caused by PAEs is mainly due to the damage of ovaries, uterus, and testes in both humans and animals, which serve as targets downstream of HPG axis. Investigating the molecular functions of the hypothalamus and pituitary gland in relation to PAEs and infertility represents a significant avenue for future research (81).

#### 4.2.2 Diagnostic technology

The identification and detection of confounding factors presents a significant challenge in the epidemiological investigation of PAEs. Currently, two primary types of interference have been identified: the first involves the co-exposure to other pollutants, such as BPA and heavy metals, which may exhibit synergistic or antagonistic interactions with PAEs, thereby obscuring or exaggerating their true effects (88). The second type of interference arises from the of exposure sources, predominantly due to variations in the use of cosmetics and plastic containers, which can result in exposure misclassification and distort the dose–response relationship. To address these challenges, it is essential to implement a three-tiered control strategy. The first tier involves precise exposure assessment (47) through the simultaneous measurement of PAEs metabolite and co-pollutants in urine. The second tier requires systematic documentation of exposure sources, including food packaging and cosmetic usage, while employing E-value analysis (89) to assess the potential impact of unmeasured confounding and to quantify the confounding effect (90). The third tier entails the enhancement of statistical models, specifically by analyzing co-exposure effects (91) using mixed exposure models, such as Bayesian Kernel Machine Regression (BKMR) or Weighted Quantile Sum (WQS) regression (92). And, the control effectiveness of the above confounding factors essentially depends on the accuracy of exposure assessment and the reliability of mechanism validation.

Consequently, in the advancement of the three-tiered control strategy, the innovation in exposure detection and mechanism research technologies, such as HPLC/MS/MS and scRNA-seq, emerges as a critical factor in overcoming existing limitations. Detection of PAEs metabolites in urine by HPLC/MS/MS is an important traditional method to evaluate the exposure level of PAEs in human (93), but recently, it is found that combined detection of PAEs metabolite levels in the urine and ovarian follicular fluids is beneficial to fully reveal the association with infertile women (94). Furthermore, single-cell RNA sequencing (scRNA-seq) technology offers a novel approach for investigating the toxicological mechanisms associated with PAEs and infertility. This technique not only elucidates the molecular pathways activated in cells following PAE exposure, such as oxidative stress, inflammatory responses, and apoptosis, but also enables the examination of the specific reactions to PAEs of various cell types over different exposure durations. These are crucial for deepening our understanding of the impact of PAEs on cellular development and the molecular mechanisms that regulate the reproductive system (95, 96) and help us explore new detection methods.

#### 4.2.3 Treatment

Avoiding exposure to PAEs and antioxidant therapy are effective measures to treat infertility caused by PAEs. Recent researches have shown that mitochondrial damage caused by ROS in male germ cells can be inhibited by regulating the Nrf 2/ARE antioxidant pathway (97, 98). Mangiferin alleviates testicular damage in rats by regulating its signaling and downregulating NF-κB signaling, thereby mediating oxidative stress, apoptosis and steroidogenesis (98, 99). Based on the above pathogenesis, it is essential to undertake a comprehensive investigation into the potential effectiveness of antioxidant therapy in alleviating the adverse impacts of PAEs on reproductive health. Such research could contribute to the development of innovative prevention and treatment approaches.

## 5 Limitation

However, there are some limitations in our study. The main points are as follows: I. The study exclusively utilized the Web of Science (WOS) database for data retrieval, we should consider integrating additional databases such as Scopus, PubMed, and Google Scholar to collect the data to in the future. II. In addition, the data collection process is restricted to articles published in the English language. These may adversely affect the integrality of the research findings. III. The published years of highly cited references or burst words are a little earlier, so the references in this paper are not all the latest, but this does not affect the prediction of research hotspots and frontiers. IV. Ignoring non-journal literature: Conference papers, technical reports, preprints, and patents are often not included, leaving out clues about technical methods and emerging trends. V. The inconsistency of the expression of the same term leads to the bias of literature aggregation. VI. Bibliometrics only counts the number of publications/citations, and cannot identify methodological flaws or low-quality studies. VII. The lack of source data in the sub-literature (incomplete information on institutional affiliation and fund funding) affects the cooperation network and policy-driven analysis. VII. There are limitations to using the H-index to measure the academic productivity and impact (Low sensitivity to highly cited papers, hindering recognition of breakthrough contributions; Irreversible time-dependent accumulation, diminishing the weight of recent scholarly output; Insufficient cross-disciplinary and career-stage discriminative power, leading to biased comparisons; Exclusion of non-h-core papers and citations).

Addressing these issues and integrating multidimensional indices is essential for to develop a more robust academic evaluation framework for studies on PAEs and infertility in future. Nonetheless, the intrinsic limitations present in the aforementioned raw data are unavoidable. This bibliometric study has endeavored to improve the analysis to the fullest extent possible.

## 6 Conclusion

PAEs are characterized by their pervasive presence and exposure, which exerting considerable effects on both environmental and human health. Notably, the prevalence of infertility has been increasing in contemporary society. Consequently, it is imperative to conduct an analysis of the existing research on PAEs and infertility to identify potential research trajectories and emerging issues of interest. This bibliometric analysis consolidates extant research to delineate knowledge trajectories and prioritize future investigations. Three fundamental challenges require urgent resolution:

- Unresolved research questions.
  1. Chronic reproductive toxicity data for long-term, low-dose, and multi-PAE mixture exposures remain unquantified, creating significant gaps in risk assessment.
  2. The synergistic or antagonistic effects of PAEs in conjunction with co-pollutants on reproductive outcomes are inadequately characterized due to methodological limitations.
  3. Causal inference in human studies is significantly constrained by the predominant reliance on cross-sectional epidemiological designs.
- Critical knowledge gaps three interconnected knowledge deficiencies hinder scientific progress.
  1. Mechanisms of PAE-induced transgenerational epigenetic dysregulation—particularly how DNA methylation and histone modifications alter germ cell development and facilitate intergenerational transmission—require systematic elucidation.
  2. The molecular targets and signaling pathways underlying cellular reproductive toxicity remain poorly defined.
  3. A translational gap exists, as evidence from rodent studies on hypothalamic–pituitary-gonadal (HPG) axis disruption shows minimal validation in humans.
- Proposed research agenda.

To address unresolved questions, we prioritize the following objectives:

1. Investigating the transgenerational dysregulation of PAEs and infertility (e.g., transgenerational dysregulation of imprinting genes).
2. Establishing chronic low-dose exposure models for cumulative risk assessment.
3. Implementing prospective cohorts with biomarker-based exposure metrics and confounder control.

To bridge existing knowledge gaps, critical actions include:

1. Employing multi-omics approaches (e.g., RNA-Seq, sc RNA-seq and HPLC/MS/MS) editing to map toxicity pathways underlying these mechanisms.
2. Developing interaction models for polychemical exposures.
3. Fostering interdisciplinary collaborations to enhance the validation processes in human studies, thereby systematically addressing the translational gap between rodent models and their applications in humans.

In conclusion, we hope our bibliometric analysis can serve to elucidate research trends while also assisting researchers in focusing on these prevailing research hotspots to offer guidance for future investigations.

## Data Availability

The original contributions presented in the study are included in the article/Supplementary material, further inquiries can be directed to the corresponding authors.

## Footnotes

^1^ www.webofscience.com

^2^ https://www.bibliometrix.org
