# Supplementary material for: Research trends and hotspots of infertility and phthalate esters: a bibliometric and visualization analysis from 2001 to 2024
Source: Front Med (Lausanne). 2025 Aug 29;12:1563179. doi: 10.3389/fmed.2025.1563179 (PMC12425914; doi:10.3389/fmed.2025.1563179)
Supplement: Supplementary file 1 [file Table_1.docx]

**Supplementary Table 1 . Top 10 co-cited references contributing to publications.**

| **Rank** | **Authors** | **Title** | **Content** | **Journals** | **DOI** | **Citations** |
| --- | --- | --- | --- | --- | --- | --- |
| 1 | Skakkebaek NE, Rajpert-De Meyts E, Main KM | Testicular dysgenesis syndrome: an increasingly common developmental disorder with environmental aspects: Opinion | This article summarizes existing evidence supporting a new concept that poor semen quality, testis cancer, undescended testis and hypospadias are symptoms of one underlying entity, the testicular dysgenesis syndrome (TDS). | Human Reproduction | 10.1093/humrep/16.5.972 | 137 |
| 2 | Fisher JS, Macpherson S, Marchetti N, Sharpe RM | Human “testicular dysgenesis syndrome”: a possible model using in-utero exposure of the rat to dibutyl phthalate. | A rat model of in utero DBP exposure (500 mg/kg/day, GD13-21) was established for the first time to fully mimic human TDS. | Human Reproduction | 10.1093/humrep/deg273 | 121 |
| 3 | Swan SH, Main KM, Liu F, Stewart SL, Kruse RL, Calafat AM, et al | Study for Future Families Research Team. Decrease in anogenital distance among male infants with prenatal phthalate exposure. Environ Health Perspect | For the first time in humans, environmental phthalate exposure is shown to disrupt male reproductive development through anti-androgenic effects (reduced AGD, higher cryptorchidism), replicating animal model mechanisms | Environ Health Perspect | 10.1289/ehp.8100 | 88 |
| 4 | Gray LE Jr, Ostby J, Furr J, Price M, Veeramachaneni DN, Parks L | Perinatal Exposure to the Phthalates DEHP, BBP, and DINP, but Not DEP, DMP, or DOTP, Alters Sexual Differentiation of the Male Rat | Phthalates DEHP and BBP demonstrated equivalent anti-androgenic potency in disrupting dose-dependent male sexual differentiation in prenatal rats (0.75 g/kg/day, GD14-PND3), while DINP was 10-fold less active and DOTP/DEP/DMP ineffective." | Toxicological Sciences | 10.1093/toxsci/58.2.350 | 84 |
| 5 | Parks LG, Ostby JS, Lambright CR, Abbott BD, Klinefelter GR, Barlow NJ, et al | The plasticizer diethylhexyl phthalate induces malformations by decreasing fetal testosterone synthesis during sexual differentiation in the male rat. | During the critical window of reproductive tract differentiation, DEHP exposure (750 mg/kg/day) reduces fetal testosterone to female levels, thereby disrupting male sexual differentiation in rats | Toxicol Sci | 10.1093/TOXSCI/58.2.339 | 81 |
| 6 | Mahood, I. K., Hallmark, N., McKinnell, C., Walker, M., Fisher, J. S., Sharpe, R. M | Abnormal Leydig Cell Aggregation in the Fetal Testis of Rats Exposed to Di (n-Butyl) Phthalate and Its Possible Role in Testicular Dysgenesis | Fetal Leydig cell aggregation is the core initiator of DBP-induced TDS, which provides a pathological model of fetal cell interaction for human testicular dysplasia. | Endocrinology | 10.1210/en.2004-0671 | 66 |
| 7 | Mylchreest E, Wallace DG, Cattley RC, Foster PM | Dose-dependent alterations in androgen-regulated male reproductive development in rats exposed to Di(n-butyl) phthalate during late gestation | Short-term prenatal exposure (10 days) of rats can cause irreversible male reproductive system damage ≥ 100 mg/kg/d, and 500 mg/kg/d can induce pretumor lesions in the testicles. | Toxicol Sci | 10.1093/toxsci/55.1.143. | 56 |
| 8 | Silva MJ, Barr DB, Reidy JA, Malek NA, Hodge CC, Caudill SP, et al | Urinary levels of seven phthalate metabolites in the U.S. population from the National Health and Nutrition Examination Survey (NHANES) 1999-2000. | This first systematic characterization of phthalate exposure in the US population revealed:  Widespread exposure to MEP, MBP, MBzP, and MEHP.  Population disparities:  Racial: African Americans exhibited significantly higher MEP levels than Mexican Americans and non-Hispanic whites.  Age: Children showed significantly elevated MBP/MBzP/MEHP exposure but lower MEP than adults.  Gender: Females had significantly higher MEP/MBzP  Low-exposure compounds: Minimal detection of MINP/MCHP/MOP suggests limited exposure to DINP/DCHP/DOP or differential metabolism. | Environ Health Perspect. | 10.1289/ehp.6723. | 56 |
| 9 | Main KM, Mortensen GK, Kaleva MM, Boisen KA, Damgaard IN, Chellakooty M, et al | Human breast milk contamination with phthalates and alterations of endogenous reproductive hormones in infants three months of age | Exposure to PAEs in breast milk may interfere with early reproductive hormone balance in male infants, consistent with impaired testicular stromal cell function observed in animal experiments, supporting evidence that perinatal PAEs affects masculinization in humans. | Environ Health Perspect | 10.1289/ehp.8075 | 54 |
| 10 | Mylchreest E, Sar M, Wallace DG, Foster PM | Fetal testosterone insufficiency and abnormal proliferation of Leydig cells and gonocytes in rats exposed to di(n-butyl) phthalate | DBP (500 mg/kg/d, GD12-21) interferes with male reproductive development through the triple pathway of testosterone synthesis inhibition-stromal cell compensatory proliferation-Sertoli cell dysfunction, providing a non-anti-androgenic mechanism model for testicular hypoplasia syndrome (TDS). | Reprod Toxicol. | 10.1016/s0890-6238(01)00201-5 | 53 |
